# Measuring Integrated Novel Dimensions in Neurodevelopmental and Stress-Related Mental Disorders (MIND-SET): Protocol for a Cross-sectional Comorbidity Study From a Research Domain Criteria Perspective

**DOI:** 10.2196/31269

**Published:** 2022-03-29

**Authors:** Philip van Eijndhoven, Rose Collard, Janna Vrijsen, Dirk E M Geurts, Alejandro Arias Vasquez, Arnt Schellekens, Eva van den Munckhof, Sophie Brolsma, Fleur Duyser, Annemiek Bergman, Jasper van Oort, Indira Tendolkar, Aart Schene

**Affiliations:** 1 Department of Psychiatry Donders Institute for Brain, Cognition and Behaviour Radboud University Nijmegen Netherlands; 2 Pro Persona Mental Health Care Depression Expertise Centre Nijmegen Netherlands; 3 Nijmegen Institute of Scientist-Practitioners in Addiction Radboud University Nijmegen Netherlands; 4 LVR-Klinikum Essen Department of Psychiatry and Psychotherapy University Hospital Essen Essen Germany

**Keywords:** psychiatry, mental health, psychiatric disorders, neuropsychology, stress, comorbidity

## Abstract

**Background:**

It is widely acknowledged that comorbidity between psychiatric disorders is common. Shared and diverse underpinnings of psychiatric disorders cannot be systematically understood based on symptom-based categories of mental disorders, which map poorly onto pathophysiological mechanisms. In the Measuring Integrated Novel Dimensions in Neurodevelopmental and Stress-Related Mental Disorders (MIND-SET) study, we make use of current concepts of comorbidity that transcend the current diagnostic categories. We test this approach to psychiatric problems in patients with frequently occurring psychiatric disorders and their comorbidities (excluding psychosis).

**Objective:**

The main aim of the MIND-SET project is to determine the shared and specific mechanisms of neurodevelopmental and stress-related psychiatric disorders at different observational levels.

**Methods:**

This is an observational cross-sectional study. Data from different observational levels as defined in the Research Domain Criteria (genetics, physiology, neuropsychology, system-level neuroimaging, behavior, self-report, and experimental neurocognitive paradigms) are collected over four time points. Included are adult (aged ≥18 years), nonpsychotic, psychiatric patients with a clinical diagnosis of a stress-related disorder (mood disorder, anxiety disorder, or substance use disorder) or a neurodevelopmental disorder (autism spectrum disorder or attention-deficit/hyperactivity disorder). Individuals with no current or past psychiatric diagnosis are included as neurotypical controls. Data collection started in June 2016 with the aim to include a total of 650 patients and 150 neurotypical controls by 2021. The data collection procedure includes online questionnaires and three subsequent sessions with (1) standardized clinical examination, physical examination, and blood sampling; (2) psychological constructs, neuropsychological tests, and biological marker sampling; and (3) neuroimaging measures.

**Results:**

We aim to include a total of 650 patients and 150 neurotypical control participants in the time period between 2016 and 2022. In October 2021, we are at 95% of our target.

**Conclusions:**

The MIND-SET study enables us to investigate the mechanistic underpinnings of nonpsychotic psychiatric disorders transdiagnostically. We will identify both shared and disorder-specific markers at different observational levels that can be used as targets for future diagnostic and treatment approaches.

## Introduction

### Background

It is widely acknowledged that comorbidity between psychiatric disorders is the rule rather than the exception [1]. Shared and diverse underpinnings of psychiatric disorders cannot be systematically understood based on symptom-based categories of mental disorders, which map poorly onto pathophysiological mechanisms. In the Measuring Integrated Novel Dimensions in Neurodevelopmental and Stress-related Mental Disorders (MIND-SET) study, we take advantage of concepts of comorbidity that transcend the current diagnostic categories in a naturalistic cohort of patients with frequently occurring psychiatric disorders and their comorbidities (excluding psychosis). The main objective of the MIND-SET project is to determine the shared and specific mechanisms of neurodevelopmental and stress-related psychiatric disorders at different observational levels. In the Introduction section, we will explain our approach generally and the choice of patients we will include.

### Current Approaches in Diagnosing Psychiatric Comorbidity

Comorbidity is not well covered by categorical, symptom-based diagnostic systems. The use of criteria to classify patients based on verbal report and observable behavior has substantially increased the reliability of psychiatric diagnoses, which serves its ultimate clinical goal of guiding treatment decisions [2,3]. However, the Diagnostic and Statistical Manual of Mental Disorders’s (Fifth Edition; DSM-5) descriptive and atheoretical approach encourages multiple diagnoses [4] and has contributed to a conceptualization of psychiatric disorders as distinct entities that should be treated according to clinical guidelines drafted for distinct disorders. Clinical practice shows that patients with the same diagnostic classification may require different treatments, while different disorders are often treated with the same interventions, indicating that a categorial approach may overlook both heterogeneity and transdiagnostic dimensions of psychopathology. Relatedly, a large body of research indicates that factors of risk and resilience for psychopathology are not unique for distinct disorders that are identified based on symptom criteria but commonly impact across diagnostic borders [5].

Not surprisingly in the light of the aforementioned controversy and the common dimensions, to date, no biological markers have been identified that are uniquely associated with specific disorders [6,7]. Conversely, diagnostic categories seem to link poorly to underlying neurobiological mechanisms, which may better map onto dimensional diagnostic approaches that incorporate the heterogeneity of psychiatric disorders. Searching for discrete etiology underlying categorical disorders is a dead end, considering the common comorbidity between disorders. Psychiatric disorders and their comorbidity should be more properly understood in a multidimensional, empirical framework, paving the way for new ways of understanding pathophysiological mechanisms of psychiatric disorders [8]. It requires a transdiagnostic perspective that regards psychiatric disorders as related disorders with distinct and shared underlying pathophysiological pathways. As is clearly illustrated by the focus of the MIND-SET study on highly prevalent neurodevelopmental and stress-related disorders that are separable diachronically, it also requires a life span and developmental perspective that distinguishes between trait and state characteristics of psychopathology.

### Comorbidity Between Neurodevelopmental and Stress-Related Disorders

In this cohort, we focus on commonly occurring comorbidities that present a challenge in diagnostics and treatment. Comorbidity between neurodevelopmental disorders such as autism spectrum disorder (ASD) and attention-deficit/hyperactivity disorder (ADHD) and stress-related disorders such as mood, anxiety, and substance use disorders is common in clinical practice [9]. Notably, comorbidity may also occur across the lifespan, suggesting a pleiotropic genetic background of common psychiatric disorders. Comorbidity is more prevalent than would be expected by chance alone, indicating that neurodevelopmental disorders may share pathophysiological mechanisms with stress-related disorders or pose a risk factor for these disorders over time. Comorbidity is associated with a higher level of functional impairment and a poorer mental health outcome [10]. At the clinical level, psychiatric comorbidity raises several questions related to complicated recognition and diagnosis, and poses therapeutic dilemmas about the most optimal treatment strategy for particular comorbidities [11]. Are depressive symptoms in someone with an ASD comparable to depressive symptoms in someone with ADHD or someone without a developmental disorder? Additionally, at the pathophysiological level, are these depressive symptoms related to, for example, biases in information processing, comparable to negative biases in major depressive disorder (MDD) without an ASD, which can be targeted with interventions such as cognitive behavioral therapy, or should treatment for the comorbid condition be modified, and if so, how? How well is someone with ASD able to recognize and verbalize their mood symptoms, and how does this impact the diagnostic procedure and the treatment choice and course? Additionally if the recognition of mood symptoms is compromised, for example, when a patient shows alexithymia, how does this affect their vulnerability to stress? For ADHD, related questions arise, such as how to distinguish core attentional deficits from concentration problems related to depression, or when do symptoms of emotional dysregulation, which are frequently observed in ADHD but not part of the formal criteria, substantiate a separate diagnosis? If so, what are the therapeutic consequences, if any? Currently, we treat comorbid depression and autism or ADHD mostly as solid entities that receive separate treatments while they may share neurobiological mechanisms that may demand different targets for treatment.

### Comorbidity Within the Research Domain Criteria Framework

**Figure 1:**
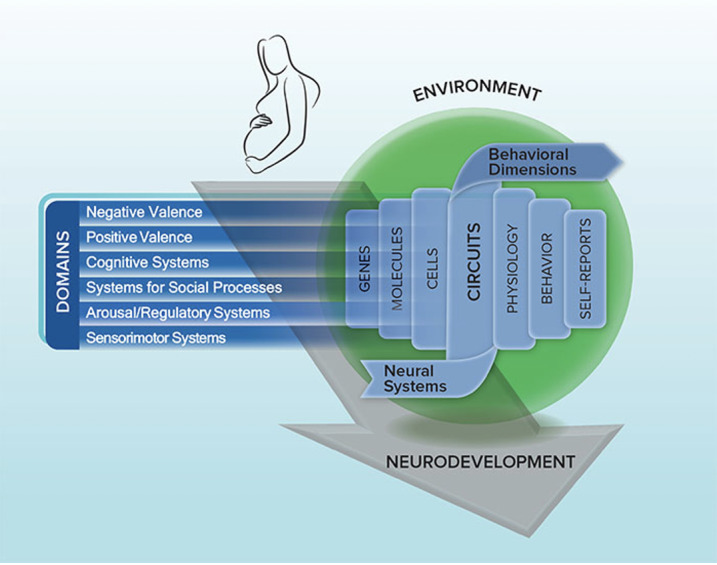
An overview of the research domain criteria framework.

High comorbidity among supposedly distinct classifications motivated the development of dimensional systems to characterize the complexity of psychiatric illness [12,13]. Trying to overcome the limitations of categorical descriptive classifications, we hence link to the Research Domain Criteria (RDoC) to study the comorbidity of neurodevelopmental and stress-related disorders (see Figure 1). The RDoC offers a research framework for understanding mental disorders in terms of varying degrees of dysfunction along basic dimensions of biological systems that have been elucidated by neuroscience. Its focus on transdiagnostic mechanisms of mental disorders is rooted in a matrix with different functional domains and within domain constructs across multiple units of analysis. Brain circuits have a central place in the units of analysis, as mental disorders are primarily regarded as disorders of the brain, which can be identified with the methods of clinical neuroscience [8]. The ultimate goal of the RDoC is to find biosignatures that on the one hand improve current diagnostic approaches [14] and on the other hand help to understand the working mechanisms of existing therapeutics and serve as targets for new treatments.

Six functional systems are identified that serve the basic motivational and adaptive needs of an organism: the negative and positive valence systems, cognitive systems, arousal and regulatory systems, social processes, and sensorimotor systems. The negative valence system directs responses to aversive stimuli or contexts, whereas the positive valence system addresses such responses to positive situations. The cognitive system contains various cognitive processes such as memory and cognitive control, whereas social processes mediate the responses to interpersonal settings. Arousal and regulatory systems include processes that are responsible for the activation of neural systems within certain contexts, as well as homeostatic regulation. Sensorimotor systems are involved in motor behaviors. Each domain contains up to seven constructs such as “acute threat” and “loss” in the negative valence system and “affiliation and attachment” and “perception and understanding of self” in the social processes system. These constructs and domains are to be analyzed with different methods and at different units of analysis: from a genetic, molecular, or cellular level to neural, or brain circuitry, and further to the physiological and behavioral level, onward to the level of self-report and paradigms.

### Data-Driven Approaches

In the light of the different levels within the RDoC framework, we aim to approach psychiatric comorbidity by data-driven approaches that are not constrained by the clinical categories. Moreover, as working principally from the RDoC perspective means working back and forth through different domains and analysis units (eg, linked independent component analysis [LICA]), we will aim to find cross-domain links with data-driven procedures and, in the end, assess the relation to clinical categories, including the descriptive comorbidities.

MIND-SET, our cross-sectional cohort study, has to be understood as a step toward understanding comorbidity from an RDoC perspective by including patients classified with neurodevelopmental disorders with an early age of onset (ASD: 1-5 years; ADHD: 5-12 years) or stress-related disorders with, on average, an adult age of onset. We include patients with at least one of these broadly used classifications, aiming to study underlying shared and distinct mechanisms. MIND-SET does not involve longitudinal changes directly (eg, improvement of prognosis through interventions) in our patients, which is the step to be taken to leverage these insights to clinical practice and which will be addressed by planned follow-up studies. The advanced understanding of comorbidity will help to progress toward innovative ideas about new therapeutic approaches that in the end will hopefully change clinical practice for patients with a multiplicity of symptoms.

### Study Aims and Outline

The main objective of the MIND-SET study is to determine the shared and specific mechanisms of neurodevelopmental and stress-related psychiatric disorders at different observational levels to gain insight in the comorbidity of the most common nonpsychotic disorders (ie, neurodevelopmental and stress-related disorders).

We will realize this aim by adopting a dimensional approach focusing on dysfunction related to stress-related (mood, anxiety, and substance use disorders) and neurodevelopmental (autism, ADHD) disorders. This will allow us to investigate connections between different units of analysis (connect symptoms with underlying circuits) and derive profiles that improve current understanding of comorbidity and ultimately can lead to better treatment.

## Methods

### Design

The MIND-SET study is an observational, cross-sectional study, in which data from different observational levels according to the RDoC units of analysis (genetics, physiology, neuropsychology, system-level neuroimaging, behavior, self-report, and experimental neurocognitive paradigms) are collected over four time points for patients with neurodevelopmental and stress-related disorders and neurotypical controls.

### Setting

The MIND-SET study is mainly executed at the outpatient unit of the psychiatric department of the Radboud University Medical Center (Radboudumc), Nijmegen, the Netherlands. The department specializes in the diagnosis and treatment of neurodevelopmental disorders and stress-related disorders in adults, with a special attention and expertise for psychiatric comorbidity and combined psychiatric and somatic pathology. Inpatients who are able to be investigated can also participate in the study.

### Population

#### Patients

##### Inclusion Criteria

Included are adult (aged ≥18 years) psychiatric patients with a clinical diagnosis of a stress-related disorder (mood disorder, anxiety disorder, or substance use disorder) or a neurodevelopmental disorder (ASD or ADHD).

##### Exclusion Criteria

Patients with diseases of the central nervous system resulting in (permanent) sensorimotor or (neuro)cognitive impairments, a current psychosis, a full-scale IQ estimate <70, inadequate command of the Dutch language, or who are mentally incompetent to give informed consent are excluded from participation. With regard to ASD, our exclusion criteria implicate that we only investigate patients with high functioning autism, without intellectual disability and without mutism. Additional exclusion criteria for the magnetic resonance imaging (MRI) session are metal objects in the body (excluding dental fillings), ferromagnetic implants or pacemakers, jewelry or piercings that cannot be removed, brain surgery, epilepsy, claustrophobia, pregnancy, and self-declared inability to lie still for more than 1 hour.

#### Neurotypical Control Participants

Individuals with no current or past psychiatric diagnosis are included. Possible eligible individuals are approached via databases of the department’s previous studies; advertisement in newspapers, social media, and websites; and via the research participation system of the Radboud University Faculty of Social Sciences (SonaSystem), as well as verbally through the researchers’ own networks. The absence of lifetime psychiatric diagnoses is assessed via a telephone screening interview, using the same diagnostic measurement instruments as described in the following section for the patient sample.

### Procedure

The data collection procedure includes an online assessment and three subsequent sessions that are planned within 1 month:

- Online assessment: Online self-report questionnaires assessing demographics, symptomatology, and functioning
- Session 1: Standardized clinical examination, physical examination, and blood sample
- Session 2: Psychological constructs, behavioral tasks, neuropsychological tests, and biological markers
- Session 3: Neuroimaging measures

The procedure for each part is briefly described in the following sections. An overview is given in Table 1, including the full names of the measurement instruments used. In the last column of Table 1, we categorize the data according to the six units of analyses as proposed by the RDoC (self-report, behavior, physiology, circuits, cells, and molecules).

**Table 1:** Data collection of the MIND-SET study: topics and instruments^a^.

Topic		Assessment	Unit of analysis^b^	Domain
**Preassessment**				
	Demographic factors	Demographics standard questionnaire	Self-report	General
	Psychiatric disorders in family	FIGS^c^	Self-report	General
	ADHD^d^ screening	ASRS^e^	Self-report	Cognitive
	ADHD symptom severity	CAARS^f^	Self-report	Cognitive
	Autistic traits	AQ-50^g^	Self-report	Social processes
	Depressive symptoms	IDS-SR^h^	Self-report	Negative valence
	Anxiety sensitivity	ASI^i^	Self-report	Negative valence
	Personality traits	PID-5-SF^j^	Self-report	General
	General health	SF-20^k^	Self-report	General
	Disability	WHO-DAS 2.0^l^	Self-report	General
	Quality of life, health related	OQ-45^m^	Self-report	General Positive valence
**Session 1: clinical examination**				
	Psychiatric diagnosis: structured clinical interviews	Neurodevelopmental disorders ADHD: DIVA^n,o^ Autism: NIDA^o,p^ Stress-related disorders Mood and Anxiety disorders: SCID-I^q^ Substance related disorder: MATE-Crimi^r^	Self-report/behavior	General
	Somatic diagnosis	Self-report questionnaire presence of somatic disease (CBS^s^)	Self-report	General
	Medication use	Medication verification	Molecules	General
	Physical examination	Height and weight Pulse rate and blood pressure (in lying and standing position) Visual acuity	Behavior/physiology	General
	Biological marker (I)	Blood sample	Molecules Cells	General
**Session 2: behavioral session**				
	Biological markers (II)	Feces microbiome Cortisol from hair sample Saliva cortisol Heart and respiration rate during stress induction in the scanner	Molecules Cells	Arousal and regulatory
	Trauma history	NEMESIS^t^-childhood trauma questionnaire	Self-report	General
	Eating behavior	Food intake: TACTICS^u^	Self-report	General
	Psychological constructs: alexithymia, behavioral regulation, repetitive thoughts	TAS-20^v^ BRIEF-A^w^ PTQ^x^	Self-report	Social processes Cognitive Negative valence
	Cognitive bias: attention bias, attention focus, memory bias, and self-referent encoding task	Noninvasive computer-mounted beam eye-tracking system Pictures of faces with different expressions (plus subsequent emotion-recognition task) Recognition of stimuli presented during the attention bias task Self-referent encoding task NB. Mood is assessed between every (sub)task and motivation after the SRET^y^ using visual analogue scales	Behavior	Cognitive systems Negative valence systems
	Executive functioning: prepotent response inhibition, interference control, updating, shifting, and reversal learning	Go no-go (from TAP 2.3^z^) Incompatibility (Simon effect; from TAP 2.3) Spatial working memory (from CANTAB^aa^) Intraextra dimensional set shift (from CANTAB) Reversal learning task	Behavior	Cognitive systems Positive valence
	Intelligence	IQ estimation	Behavior	Cognitive
	Underachievement	Alertness (from TAP 2.3)	Behavior	Arousal and regulatory
**Session 3: neuroimaging session**				
	Brain structure and brain function: salience network, default mode network, and central executive, and stress-induced network changes	MRI^ab^ T1 scan DTI^ac^ Emotional face matching task Resting state fMRI^ad^ connectivity rs-fMRI^ae^ during/after aversive vs neutral movie	Neural circuits/physiology	All domains Social processes Negative valence

^a^For a more detailed description of data collection: see Multimedia Appendix 1.

^b^We use the 6 units of analysis of the initiative Research Domain Criteria: genes, molecules, cells, neural circuits, physiology, and behavior.

^c^FIGS: Family Interview for Genetic Studies.

^d^ADHD: attention-deficit/hyperactivity disorder.

^e^ASRS: Adult ADHD Self-Report Scale.

^f^CAARS: Conners’ Adult ADHD Rating Scale.

^g^AQ-50: Autism Spectrum Quotient-50.

^h^IDS-SR: Inventory of Depressive Symptomatology–Self Rating.

^i^ASI: Anxiety Sensitivity Index.

^j^PID-5-SF: Personality Inventory for DSM-5–Short Form.

^k^SF-20: Short Form-20.

^l^WHO-DAS 2.0: World Health Organization Disability Assessment Schedule 2.0.

^m^OQ-45: Outcome Questionnaire.

^n^DIVA: Diagnostic Interview for Adult ADHD.

^o^DIVA and NIDA are only carried out in case of positive screening (ASRS>3 or AQ>25) or clinical judgement.

^p^NIDA: Dutch Interview for Autism Spectrum Disorders in Adults.

^q^SCID-I: Structured Clinical Interview for DSM-IV Axis I Disorders; section A,B,C,D,F.

^r^MATE-Crimi: Measurements in the Addictions for Triage and Evaluation and Criminality.

^s^CBS: Central Bureau voor Statistitiek.

^t^NEMESIS: Netherlands Mental Health Survey and Incidence Study.

^u^TACTICS: Translational Adolescent and Childhood Therapeutic Interventions in Compulsive Syndromes.

^v^TAS-20: Toronto Alexithymia Scale-20.

^w^BRIEF-A: Behavior Rating Inventory Executive Function–Adult.

^x^PTQ: Perseverative Thinking Questionnaire.

^y^SRET: self-referent encoding task.

^z^TAP 2.3: Testbatterie zur Aufmerksamkeitsprüfung Version 2.3.

^aa^CANTAB: Cambridge Neuropsychological Test Automated B.

^ab^MRI: magnetic resonance imaging.

^ac^DTI: diffusion tensor imaging.

^ad^fMRI: functional MRI.

^ae^rs-fMRI: resting station fMRI.

#### Online Assessment

##### Questionnaires

All patients referred to the outpatient psychiatric department receive log-in details for an online questionnaire batch at home. They are asked to fill out the questionnaires within 21 days before their first appointment. If preferred, a paper copy is sent to their home address. The questionnaires assess demographics; psychiatric disorders in the family; symptoms of ADHD, depression, and anxiety; and autistic and personality traits. Two questionnaires are also used as screening instruments for autism and ADHD. Finally, questionnaires on general health, disability or functional limitations, and quality of life are included. Summary and subscale scores derived from these questionnaires are made available before the clinical examination session to inform the clinician about the possible involvement of neurodevelopmental and stress-related disorders, personality problems, and functional status.

#### Session 1: Clinical Examination

##### Diagnostics

During a 3-hour clinical examination at the psychiatric department, patients undergo a psychiatric, biographical, and somatic anamnesis; medication verification; review of treatment history; structured clinical interviews; a physical examination; and a questionnaire assessment of the presence of somatic diseases. Examinations are conducted by well-trained clinicians: psychiatrists, psychologists, supervised psychiatric residents, supervised nurse practitioners, and supervised psychology interns. At the end of the examination, the senior clinician assesses eligibility based on the DSM-5 classification (see Measures section) and completes the written informed consent procedure. The patient consents to the use of their questionnaire data for research, the use of their diagnostic data for research, and participation in the next sessions of the study. After giving informed consent, blood sampling is executed and appointments for sessions 2 and 3 are scheduled to take place as soon as possible and ultimately within 90 days.

#### Session 2: Behavioral Assessment

##### Biomarkers

First, patients receive a package and instructions for the collection of a feces sample at home. They are instructed on how to return this package by mail. Next, hair samples are taken for cortisol measurement.

##### Questionnaires and Neuropsychology

First, patients undergo a neuropsychological assessment (~120 minutes), including a pen and paper task and several computer tasks including an eye-tracking task. The test battery is administered by a trained research assistant. Participants are then required to fill out questionnaires (~20 minutes) assessing trauma history, food intake, and three psychological constructs (alexithymia, repetitive thoughts, and behavioral regulation). A research assistant is available for assistance.

#### Session 3: Neuroimaging

This final session (180 minutes) is scheduled in the afternoon to account for the diurnal changes in cortisol levels at the Centre for Cognitive Neuroimaging of the Donders Institute for Brain, Cognition and Behavior in Nijmegen. It starts with an acclimatization period during which participants fill in questionnaires about current mood state and recent medication changes, and watch a relaxing nature documentary. Hereafter, they are prepared for the MRI scanner and undergo different imaging paradigms, including a T1 structural MRI, diffusion tensor imaging, functional MRI (fMRI) during an emotion-recognition task, and a baseline resting state fMRI. It continues with resting state fMRI after a neutral and a highly aversive movie clip, meant as a brief stress induction procedure. During the whole imaging session, physiological data are collected, such as heart and respiration rate, and saliva for cortisol and alpha-amylase measurement is collected at different time points in addition to assessments of mood, stress level, and other emotions. The neuroimaging session ends with a short debriefing procedure.

### Ethics Approval and Consent to Participate

#### Regulation Statements

The MIND-SET study has been approved by the local medical ethical committee (Commissie Mensgebonden Onderzoek Arnhem-Nijmegen). After verbal and written information about the study that they receive at home, eligible participants are approached by their care provider for participation in the study. If interested, they sign an informed consent form. Written informed consent is provided for clinical data use and data collection. In the course of the study, a yearly data monitoring is conducted with a local monitor of the Radboudumc Nijmegen.

All diagnostic interviews, neuropsychological measures, physiological measures, and neuroimaging measures are conducted by extensively trained clinicians and research assistants. All clinicians received diagnostic interview training from certified and experienced trainers. All research professionals conducting the neuropsychological tests received extensive training by neuropsychological testing experts.

#### Compensation

Participants are compensated with travel costs for the data collection sessions, and the controls are as well paid a small fee for their participation according to the guidelines of the medical ethical committee: €10 (US $11) per hour and €66 (US $73) in total.

### Measures

Multimedia Appendix 1 offers a complete description of the specific instruments and measures. Here, we focus on the levels of psychopathology, neuropsychology, and brain circuits.

#### Descriptive Psychopathology Level

Psychopathology is addressed along a continuum ranging from the syndrome or disorder level (Diagnostic and Statistical Manual of Mental Disorders [Fourth Edition; DSM-IV] and DSM-5) to the disorder-related symptomatic level and to the transdiagnostic dimensional level.

Neurodevelopmental disorders are assessed in case of either positive screening or based on clinical judgment by diagnostic interviews. For screening on ASD traits, we use the Autism Spectrum Quotient (AQ-50) [15]. When a patient scores positive on this instrument (50 items, cutoff >25), we next use the Dutch Interview for the Diagnosis of ASD in Adults (NIDA) [16] to diagnose ASD according to the DSM-5. Regarding ADHD, we use the World Health Organization Adult ADHD Self-report Scale short version for screening [17]. In case of positive screening (6 items, cutoff >3), we subsequently conduct the Diagnostic Interview for ADHD in Adults (DIVA) [18] to diagnose ADHD according to the DSM-IV. Both the DIVA and NIDA are completed in the presence of a partner or family member of the patient (if available) to ascertain information retrospectively and collaterally on a broad range of symptoms in childhood and adulthood. The Structured Clinical Interview for DSM-IV Axis I Disorders [19] is used to diagnose mood (depression and anxiety) disorders and to exclude psychotic disorders. To diagnose substance-related disorders according to the DSM-5, we use an adapted version of the Measurements in the Addictions for Triage and Evaluation and Criminality [20].

A set of questionnaires provide measures of depression (Inventory of Depressive Symptomatology), anxiety (Anxiety Sensitivity Index), and ADHD symptoms (Conners’ Adult ADHD Rating Scale) not only to provide dimensional measures that fit with the syndromes that are our primary diagnoses but also to assess comorbidity at the symptomatic level in the context of other diagnostic categories. We use the Personality Inventory for DSM-5 to assess personality trait domains including negative affect, detachment, antagonism, disinhibition, and psychoticism, and the AQ-50 to measure traits that are related to autism in adults with normal intelligence. The personality traits and autistic traits may measure overlapping domains. We have included three questionnaires that address psychological constructs that cut across syndromes and reveal transdiagnostic mechanisms important for understanding comorbidity. We include the Perseverative Thinking Questionnaire and alexithymia (Toronto Alexithymia Scale-20) and behavioral regulation (Behavior Rating Inventory Executive Function–Adult) questionnaires. In addition, a structured inventory developed for the NEMESIS (Netherlands Mental Health Survey and Incidence Study) epidemiological study assesses an individual’s trauma history before the age of 16 years, including emotional neglect or psychological, physical, and sexual abuse [21,22].

#### Neuropsychological Level

The RDoC unit behavior is operationalized by neuropsychological assessments within the domains of the negative valence systems (constructs: sustained threat, loss), positive valence systems (construct: reward learning), and cognitive systems (constructs: attention, declarative memory, cognitive control).

#### Negative Valence System

Affective neuropsychological tests assess emotional processing, and in the context of the negative valence system, we focus on several cognitive biases. We assess attentional bias for both social and nonsocial negative and positive pictures by means of a free-viewing eye-tracker task (with a noninvasive computer-mounted *beam* eye-tracking system) and a subsequent recognition task to assess memory bias during eye-tracking. Measuring eye movements during a task using an eye-tracker is regarded as a reliable measure for attentional focus [23]. As patients with autism generally show decreased attention to social information [24], we have chosen to incorporate both social and nonsocial pictures with either negative or positive valence to be able to dissociate the differential contribution of these factors on attentional processes. In addition, memory bias is tested by a computerized self-referent encoding task [25] in which participants have to indicate how characteristic different positive and negative adjectives are to them and are subsequently tested for correct recall of these adjectives after a distraction task. Visual analogue scales are used to assess mood at four different time points throughout the assessment to account for the influence of mood on performance, as well as self-reported effort on the tests afterward.

#### Positive Valence System

Within this domain, we measure the construct of reward learning. Learning can be influenced by the valence of the feedback given on the performance during the task. For example, previous studies have found reduced learning from reward in mood disorders [26-29]. We use a probabilistic reversal learning task [30-32] to examine reward and punishment sensitivity in a changing context. First, participants learn a stimulus-response relationship by trial and error, after which the stimulus-response relationship is reversed without explicit warning, and they have to change their response. Reversal learning is an important aspect of cognitive flexibility, which supports someone to adapt to changing environmental conditions including rewards [33].

#### Cognitive Systems

Impairments in emotional regulation are common in both stress-related and neurodevelopment disorders. Our aim here is to study the nature of these alterations in executive functioning by studying prepotent response inhibition, interference control, updating and shifting across stress-related and neurodevelopmental disorders to better understand the underlying mechanisms of shared symptoms such as impaired emotion regulation, rigidity, and impulsivity.

#### Brain Circuits Level

**Figure 2:**
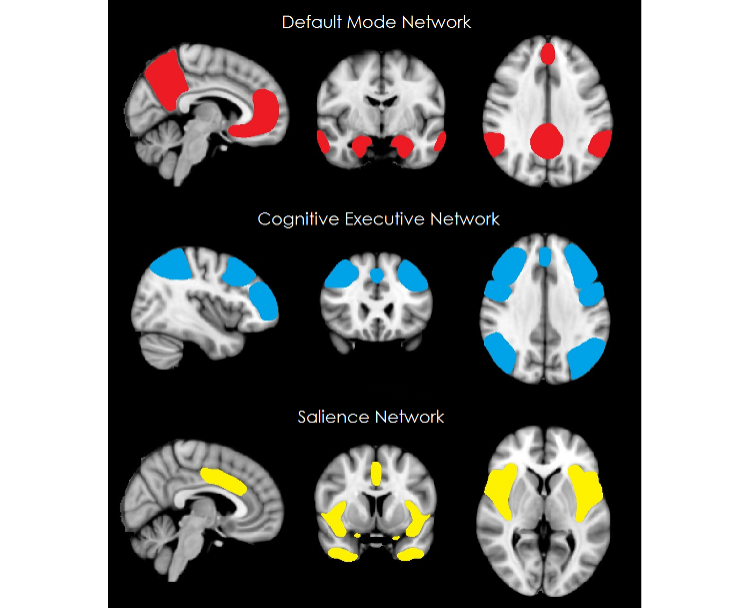
Representation of relevant resting-state networks with the default mode network depicted in red, the central executive network in blue, and the salience network in yellow.

The brain circuits level is at the core of our research design, as it bears on the hypothesis that the phenotypic, behavioral differences among psychiatric disorders can be explained by differences in the underlying neural circuitry, while downstream causal mechanisms such as genetic and epigenetic effects or environmental factors will lead to psychiatric symptoms and disorders via their disruptive effects on neural circuits. The brain is dynamically organized into functional networks of interconnected areas, which interact to perform unique brain functions. These networks can be consistently identified with functional MRI scans during the “resting-state” by calculating functional connectivity between voxels. The most relevant networks with regard to psychiatric disorders are the default mode network (DMN), involved in emotion regulation, self-reference, and obsessive ruminations [34]; the salience network, which plays a central role in emotional control [35]; and the central executive network, which is most active during cognitive tasks and is relevant for attention and working memory (see Figure 2).

Together these networks cover the most important functional domains such as top-down cognitive control, conflict signaling, salience detection, and self-referential processing that are affected in both stress-related and neurodevelopmental disorders. Small pilot studies with this approach have already demonstrated that hyperconnectivity in components of the DMN is associated with depressive symptoms such as ruminations and self-absorption, while hypoconnectivity in components of the DMN is associated with anxiety symptoms [36]. Studying the dynamics of network connectivity, in conditions of both rest and stress, allows us to disentangle fundamental pathophysiological mechanisms underlying these disorders and their shared mechanisms that are relevant for understanding comorbidity.

#### Negative Valence System

We will investigate functional networks both during resting state and during a brief stress induction procedure (acute threat paradigm). Previous research has shown that acute stress shifts the brain into a state that fosters rapid defense mechanisms [36]. Stress-related neuromodulators are thought to trigger this change by altering properties of large-scale neural populations throughout the brain. In neurotypical participants, we have shown that noradrenergic activation during acute stress results in prolonged coupling within a distributed network that integrates information exchange between regions involved in autonomic-neuroendocrine control and vigilant attentional reorienting. It remains unclear to what extent these mechanisms are altered by psychiatric diseases, thereby reflecting an acute measurement of vulnerability and disease load. Functional measures will be complemented by diffusion-weighted imaging to provide measures of structural connectivity between the networks. Further, we want to explore if dynamic functional connectivity data along the baseline-stress-recovery axis for the three distinct networks will serve to identify differences in the dynamic balance in these networks at the individual participant level and can be related to behavioral and symptom profiles.

#### Social Processes

An emotional face matching task addresses the subconstruct reception of facial communication within this domain. This paradigm engages the amygdala and an amygdala-centered network by contrasting the BOLD response during blocks of angry and fearful face stimuli with blocks with geometric shapes that consist of scrambles of the same face stimuli [37,38]. This task is commonly used as a paradigm to probe amygdala reactivity, and aberrant amygdala reactivity has been implicated in both stress-related and neurodevelopmental disorders.

### Data Analysis

#### Sample Size

This research protocol will comprise multiple studies to be conducted across multiple years. The majority of studies will estimate effects at the population level by means of parametric *t*, *F*, or chi-square tests, where empirical evidence from our and other centers suggests that typical study sizes of ~20 to 30 participants per group can be sufficient to detect relevance between group differences, given typical effect sizes across a variety of data modalities. After consulting a biostatistician, we decided that an overall sample size calculation will be of little value. Additionally, power calculations for studies with MRI are difficult and not used routinely, but here, there is also consensus that groups of ≥20 usually yield sufficient power in MRI studies to detect moderate differences in regions of interest. Based on these considerations and to have at least 20 participants per group in the broadly defined comorbid conditions, we aim to include a total of 650 patients and 150 neurotypical control participants in the time period between 2016 and 2022. In October 2021, we are at 95% of our target. Many research studies that will be conducted under this proposal will be exploratory in nature, where not much prior reference work is available. In these cases, we will use expected effect size estimates and ranges thereof generated from testing small samples in pilot studies to inform sample size calculations. In these sample size calculations, we expect that for cross-sectional analyses, with a power of 80% and an alpha of .05, we will be able to detect small differences with respect to clinical variables and questionnaires.

#### Data Handling

We will store raw and cleaned data in a digital research environment. Data is also shared with researchers via the digital research environment. A variety of analysis software and statistical programs will be used to analyze the data. Statistical analysis will be performed within, for example, SPSS (version 25; IBM Corp) and R (R Foundation for Statistical Computing; version 3.6.1). Analysis of neuroimaging data will be performed with, for example, FSL (FMRIB Software Library version 5.0) for connectivity analyses before and after stress induction, SPM12 (Statistical Parametric Mapping version 12) for the emotional face matching task, and Freesurfer (version 6.0.0) for analysis of the structural MRI and diffusion data. Data will be analyzed according to the state-of-art analyses insights and using relevant new techniques and approaches where applicable.

Digitalized diagnostic interviews are used to facilitate completeness of the diagnostic data. A data manager coordinates the data entry in the digital research environment while also checking data quality. Data archiving and creating variables and scales is part of data management. Yearly study monitoring is carried out by an independent monitor to assess adherence to the procedures and to ensure patient safety and privacy.

#### Statistical Analyses

Detailed processing and statistical methods applying to the different measures and levels are presented in Multimedia Appendix 1. We will use exploratory factor analysis within SPSS to uncover domains of functioning that transcend conventional diagnostic (DSM) boundaries and investigate shared and distinct variance that is measured by the different questionnaire and instruments at the descriptive psychopathological level. We will use parallel analysis and skree-plots to find optimal factor solution (maximum likelihood estimation, oblique rotation).

We will apply univariate statistics within the framework of the general linear models or linear mixed models to investigate differences in specific measures between different disorders and investigate relations between different measures. As an example, we will use analyses of covariance to compare different diagnostic groups on negative memory bias scores and investigate associations between negative memory bias and depression symptom severity with linear regression models. As we collect a large set of measures and perform a large number of comparisons, which carries the risk of false positives, we will only perform analyses according to a priori–specified analysis plans that are approved by the steering board of MIND-SET, and we will apply appropriate corrections for multiple comparisons. In addition, multivariate analyses can further reduce the risk of false positives.

The ultimate goal is to relate features of the different units of analysis across the different domains with multivariate methods. To exploit the multimodal, multilevel dimensions of our data, we will apply advanced statistical methods to identify relevant multivariate patterns, including machine learning, factor, and network analyses. Extracted components from the self-report, behavior, and physiological data are used as inputs in regularized canonical correlation analyses to detect connections among the different units of analysis and identify transdiagnostic patterns in the data.

LICA is a new analysis technique, which integrates different imaging modalities and link shared patterns, or so-called independent components, to interindividual differences in behavior and psychopathology (Llera et al [39]). LICA combines imaging modalities at an early stage in the analysis pipeline, rather than a post hoc combination of unimodal results at the stage of final interpretation (Groves et al [40]). LICA has not yet been used within a transdiagnostic research context.

Finally, we will adopt a normative modeling approach for mapping associations between brain function, biological and clinical measures, and behavior to estimate deviation from the normative model on a participant level. Normative modeling provides a framework to characterize patients individually in relation to normal functioning, which may be far more informative than categorical labels. This approach may help to parse the heterogeneity that is common in clinical cohorts and point to more biologically valid subtypes [41].

### Dissemination

The study results will be published in peer-reviewed journals and distributed via media outlets. We will post our preprints at bioRxiv or medRxiv, free online archives, and distribution services for unpublished preprints in the life and medical sciences. It is operated by Cold Spring Harbor Laboratory, a not-for-profit research and educational institution. By posting preprints on bioRxiv and medRxiv, MIND-SET authors are able to make their findings immediately available to the scientific community and receive feedback on draft manuscripts before they are submitted to journals. Results will further be presented at national and international congresses and meetings. Participants are notified of study progress and outcome by means of newsletters.

### Availability of Data and Materials

The data sets generated or analyzed during this study are not publicly available due to privacy reasons but are made available for researchers within the digital research environment upon reasonable request to the corresponding author and approval of the steering board of the MIND-SET study group.

## Results

We aim to include a total of 650 patients and 150 neurotypical control participants in the time period between 2016 and 2022. In October 2021, we are at 95% of our target.

## Discussion

### Transdiagnostic Approach

Psychiatric disorders and their comorbidity could be more properly understood in a multidimensional, empirical framework, adopting a transdiagnostic perspective that regards psychiatric disorders as related disorders with distinct and shared underlying pathophysiological pathways. The MIND-SET study is setup to investigate the mechanistic underpinnings of stress-related and neurodevelopmental disorders to identify both shared and disorder-specific markers at different observational levels that are based on RDoC domains. Here, we will specifically focus on the importance of studying cognitive systems and negative valence system together and at different observational levels.

Negative affect such as depressed mood and anxiety, both on a symptomatic and syndromic level, is frequently comorbid in neurodevelopmental disorders. We know for example that later in life, individuals with ASD have a four times higher lifetime prevalence of depression. Although ASD is primarily characterized by alterations in sensory sensitivity, inflexible routines, restricted interests, and deficits in social functioning or rather neurodivergent social functioning [42], about 50% of high-functioning adults diagnosed with ASD who were referred to a psychiatry department had comorbid MDD [43]. Because of the overlap of symptoms and personality characteristics (eg, rigidity), depression is often difficult to recognize in ASD and remains frequently undetected [44]. Individuals with ASD have difficulties reading their own inner states, and clinicians lack diagnostic tools and treatment options. Recognition and treatment are needed, as individuals with MDD and ASD have lower global functioning compared to individuals with ASD only.

Our understanding of MDD in neurodevelopmental disorders remains limited today, as well as our treatment options. One possibility is that negative affect results from increased levels of stress sensitivity that are related to the primary deficits, for example, increased levels of stress caused by sensory overstimulation or problems in relationships related to deficits in social cognition and flexibility [45]. ASD and ADHD are both associated with impairments in executive function, and each disorder is thought to have its specific deficits, with impairment in shifting most prominent in ASD [46], while ADHD is typically characterized by problems with behavioral inhibition [47]. Evidence suggests that impairment of executive function is an important predictor of comorbid anxiety and depression, and that specific deficits of ASD and ADHD may reveal pathways to comorbidities in these disorders [48].

Performance of executive function in ASD is thought to be related to poor regional coordination and integration of prefrontal executive processes that integrate with emotion and social circuits, reflected by aberrant patterns of connectivity with both changes of within- and between-network functional connectivity scale networks [49]. A recent data-driven approach identified three transdiagnostic subtypes of executive functioning in a large sample of children with ASD, ADHD, and neurotypical children that spanned the normal to impaired spectrum but also cut across ADHD and ASD samples. Moreover, these subtypes of executive functioning better accounted for variance in the neuroimaging data than DSM diagnoses did, highlighting the point that transdiagnostic subtypes may indeed refine current diagnostic classifications [50].

Individuals with ASD and ADHD may also be more vulnerable to depression and anxiety because they share information processing styles that are related to the susceptibility for depression and anxiety, such as biases in information processing [51]. Biases in information processing have traditionally been studied within the boundaries of diagnostic categories and have mainly been studied in affective disorders. Patients with depression show more attention toward negative information, which probably points to a difficulty to disengage from negative information [52], but in comparison with neurotypical individuals, they also show less attention to positive stimuli [53]. Negative memory bias seems to be associated with a higher level of comorbidity among psychiatric disorders [54]. Biased information processing may therefore constitute a transdiagnostic mechanism for psychopathological symptoms, which seems crucial for understanding comorbidity. This biased information processing constitutes a cognitive vulnerability that, according to Beck’s [55,56] model, is linked to the experience of adverse events during childhood, which may lead to dysfunctional cognitive schemas.

In our mechanistic approach to investigate underlying cross-domain processes to explain patterns of comorbidity across a range of neurodevelopmental and stress-related disorders, both executive functioning and emotional information processing are key mechanistic elements that may interact in specific ways across different levels of analysis. Recent neurocognitive findings suggest that problems in emotion regulation result from preferential processing of (negative) emotional information in subcortical structures, including overactivation of an amygdala-centered network and reduced prefrontal executive control to inhibit inappropriate emotions and emotion expression (eg, [57-59]). Habituation of the amygdala response may also play a role here, as it has been shown to correlate negatively with anxiety [60] and is decreased in ASD [61-63]. Both amygdala activation and habituation have been frequently used in genetic imaging studies to investigate the neural effects of genetic variants that are linked to depression, anxiety, and personality traits like neuroticism [63,64]. For example, the short allele of the serotonin transporter gen has been associated with increased risk for depression after exposure to stress, which is thought to be mediated by increased amygdala reactivity to threat [64].

Moreover, the function of covert cognitive mechanisms in several cross-disorder symptoms such as impulsivity, apathy, or alexithymia are yet unknown. Characterizing these mechanisms may allow us to identify different underlying profiles that combine executive dysfunction and emotional process biases, and could serve as targets for new treatments such as neuromodulation. A specific example, which may illustrate partly overlapping mechanisms, is a deficit in mental shifting that may be implied in preoccupied and rigid thinking that is characteristic for ASD but which is also implied in the ruminative thinking that characterizes depression. In individuals with ASD, there is some evidence that poorer executive functioning (and greater behavioral inflexibility) predicts greater anxiety and depression [48,65]. Similarly, executive deficits have been related to rumination [66] and the susceptibility to depression [57]. In addition, early life adversity may have caused enhanced corticolimbic reactivity that, in turn, leads to rumination, which is known to be a vulnerability factor for internalizing psychiatric disorders [67].

### Limitations

This study has to been understood in the light of some limitations. Although we aim for a fairly large sample size (we aim to include a total of 650 patients and 150 neurotypical control participants), specific cells of comorbidity between disorders may be low for group comparisons. Moreover, the participants are all recruited at one psychiatric center (ie, the Psychiatric Department of the Radboud University Medical Center), which specializes in the diagnosis and treatment of neurodevelopmental disorders and stress-related disorders in adults and their comorbidity, and this constitutes a form of selection bias and decreases generalizability of the study results to other populations.

## Appendix

Multimedia Appendix 1 Supplemental material.

## Abbreviations

ADHD: attention-deficit/hyperactivity disorder
AQ-50: Autism Spectrum Quotient
ASD: autism spectrum disorder
DIVA: Diagnostic Interview for Attention-Deficit/Hyperactivity Disorder in Adults
DMN: default mode network
DSM-IV: Diagnostic and Statistical Manual of Mental Disorders (Fourth Edition)
DSM-5: Diagnostic and Statistical Manual of Mental Disorders (Fifth Edition)
fMRI: functional magnetic resonance imaging
LICA: linked independent component analysis
MDD: major depressive disorder
MIND-SET: Measuring Integrated Novel Dimensions in Neurodevelopmental and Stress-Related Mental Disorders
MRI: magnetic resonance imaging
NEMESIS: Netherlands Mental Health Survey and Incidence Study
NIDA: Dutch Interview for the Diagnosis of Autism Spectrum Disorder in Adults
RDoC: Research Domain Criteria

## References

[1] van Loo HM, Romeijn J, de Jonge P, Schoevers RA (2013). Psychiatric comorbidity and causal disease models. Prev Med.

[2] Kendler KS, Aggen SH, Knudsen GP, Røysamb E, Neale MC, Reichborn-Kjennerud T (2011). The structure of genetic and environmental risk factors for syndromal and subsyndromal common DSM-IV axis I and all axis II disorders. Am J Psychiatry.

[3] Frances AJ, Widiger T (2012). Psychiatric diagnosis: lessons from the DSM-IV past and cautions for the DSM-5 future. Annu Rev Clin Psychol.

[4] First MB, Pincus HA, Levine JB, Williams JBW, Ustun B, Peele R (2004). Clinical utility as a criterion for revising psychiatric diagnoses. Am J Psychiatry.

[5] Buckholtz JW, Meyer-Lindenberg A (2012). Psychopathology and the human connectome: toward a transdiagnostic model of risk for mental illness. Neuron.

[6] Kupfer D, First M, Regier D (2002). A Research Agenda for DSM-V.

[7] Widiger TA, Samuel DB (2005). Diagnostic categories or dimensions? A question for the Diagnostic And Statistical Manual Of Mental Disorders--fifth edition. J Abnorm Psychol.

[8] Morris SE, Cuthbert BN (2012). Research Domain Criteria: cognitive systems, neural circuits, and dimensions of behavior. Dialogues Clin Neurosci.

[9] Buck TR, Viskochil J, Farley M, Coon H, McMahon WM, Morgan J, Bilder DA (2014). Psychiatric comorbidity and medication use in adults with autism spectrum disorder. J Autism Dev Disord.

[10] White SW, Simmons GL, Gotham KO, Conner CM, Smith IC, Beck KB, Mazefsky CA (2018). Psychosocial treatments targeting anxiety and depression in adolescents and adults on the autism spectrum: review of the latest research and recommended future directions. Curr Psychiatry Rep.

[11] Katzman MA, Bilkey TS, Chokka PR, Fallu A, Klassen LJ (2017). Adult ADHD and comorbid disorders: clinical implications of a dimensional approach. BMC Psychiatry.

[12] Kotov R, Krueger RF, Watson D, Achenbach TM, Althoff RR, Bagby RM, Brown TA, Carpenter WT, Caspi A, Clark LA, Eaton NR, Forbes MK, Forbush KT, Goldberg D, Hasin D, Hyman SE, Ivanova MY, Lynam DR, Markon K, Miller JD, Moffitt TE, Morey LC, Mullins-Sweatt SN, Ormel J, Patrick CJ, Regier DA, Rescorla L, Ruggero CJ, Samuel DB, Sellbom M, Simms LJ, Skodol AE, Slade T, South SC, Tackett JL, Waldman ID, Waszczuk MA, Widiger TA, Wright AGC, Zimmerman M (2017). The Hierarchical Taxonomy of Psychopathology (HiTOP): a dimensional alternative to traditional nosologies. J Abnorm Psychol.

[13] Lahey BB, Krueger RF, Rathouz PJ, Waldman ID, Zald DH (2017). Validity and utility of the general factor of psychopathology. World Psychiatry.

[14] Cuthbert BN, Insel TR (2013). Toward the future of psychiatric diagnosis: the seven pillars of RDoC. BMC Med.

[15] Baron-Cohen S, Wheelwright S, Skinner R, Martin J, Clubley E (2001). The autism-spectrum quotient (AQ): evidence from Asperger syndrome/high-functioning autism, males and females, scientists and mathematicians. J Autism Dev Disord.

[16] Vuijk R (2016). Nederlands interview ten behoeve van diagnostiek autismespectrumstoornis bij volwassenen (NIDA).

[17] Kim J, Lee E, Joung Y (2013). The WHO Adult ADHD Self-Report Scale: reliability and Validity of the Korean Version. Psychiatry Investig.

[18] Kooij J, Francken M (2010). DIVA 2.0. Diagnostic Interview for ADHD in adults (DIVA).

[19] First M, Gibbon M, Spitzer R, Williams J, Benjamin L (1997). Structured Clinical Interview for DSM-IV-TR Axis I Disorders, Research Version, Patient Edition. (SCID-I/P).

[20] Schippers GM, Broekman TG, Buchholz A, Koeter MWJ, van den Brink W (2010). Measurements in the Addictions for Triage and Evaluation (MATE): an instrument based on the World Health Organization family of international classifications. Addiction.

[21] de Graaf R, Bijl RV, Smit F, Vollebergh WAM, Spijker J (2002). Risk factors for 12-month comorbidity of mood, anxiety, and substance use disorders: findings from the Netherlands Mental Health Survey and Incidence Study. Am J Psychiatry.

[22] de Graaf R, Ten Have M, van Dorsselaer S (2010). The Netherlands Mental Health Survey and Incidence Study-2 (NEMESIS-2): design and methods. Int J Methods Psychiatr Res.

[23] Waechter S, Nelson AL, Wright C, Hyatt A, Oakman J (2013). Measuring attentional bias to threat: reliability of dot probe and eye movement indices. Cogn Ther Res.

[24] Guillon Q, Hadjikhani N, Baduel S, Rogé B (2014). Visual social attention in autism spectrum disorder: insights from eye tracking studies. Neurosci Biobehav Rev.

[25] Derry PA, Kuiper NA (1981). Schematic processing and self-reference in clinical depression. J Abnorm Psychol.

[26] Eshel N, Roiser JP (2010). Reward and punishment processing in depression. Biol Psychiatry.

[27] Robinson OJ, Cools R, Sahakian BJ (2012). Tryptophan depletion disinhibits punishment but not reward prediction: implications for resilience. Psychopharmacology (Berl).

[28] Admon R, Pizzagalli DA (2015). Dysfunctional reward processing in depression. Curr Opin Psychol.

[29] Safra L, Chevallier C, Palminteri S (2019). Depressive symptoms are associated with blunted reward learning in social contexts. PLoS Comput Biol.

[30] Swainson R, Rogers RD, Sahakian BJ, Summers BA, Polkey CE, Robbins TW (2000). Probabilistic learning and reversal deficits in patients with Parkinson's disease or frontal or temporal lobe lesions: possible adverse effects of dopaminergic medication. Neuropsychologia.

[31] Cools R, Barker RA, Sahakian BJ, Robbins TW (2001). Enhanced or impaired cognitive function in Parkinson's disease as a function of dopaminergic medication and task demands. Cereb Cortex.

[32] den Ouden HEM, Daw ND, Fernandez G, Elshout JA, Rijpkema M, Hoogman M, Franke B, Cools R (2013). Dissociable effects of dopamine and serotonin on reversal learning. Neuron.

[33] Cools R, Clark L, Robbins TW (2004). Differential responses in human striatum and prefrontal cortex to changes in object and rule relevance. J Neurosci.

[34] Gusnard DA, Akbudak E, Shulman GL, Raichle ME (2001). Medial prefrontal cortex and self-referential mental activity: relation to a default mode of brain function. Proc Natl Acad Sci U S A.

[35] Seeley WW, Menon V, Schatzberg AF, Keller J, Glover GH, Kenna H, Reiss AL, Greicius MD (2007). Dissociable intrinsic connectivity networks for salience processing and executive control. J Neurosci.

[36] Hermans EJ, van Marle HJF, Ossewaarde L, Henckens MJAG, Qin S, van Kesteren MTR, Schoots VC, Cousijn H, Rijpkema M, Oostenveld R, Fernández G (2011). Stress-related noradrenergic activity prompts large-scale neural network reconfiguration. Science.

[37] Hariri A, Mattay VS, Tessitore A, Fera F, Smith WG, Weinberger DR (2002). Dextroamphetamine modulates the response of the human amygdala. Neuropsychopharmacology.

[38] van Wingen GA, van Broekhoven F, Verkes RJ, Petersson KM, Bäckström T, Buitelaar JK, Fernández G (2008). Progesterone selectively increases amygdala reactivity in women. Mol Psychiatry.

[39] Llera A, Wolfers T, Mulders P, Beckmann CF (2019). Inter-individual differences in human brain structure and morphology link to variation in demographics and behavior. Elife.

[40] Groves AR, Smith SM, Fjell AM, Tamnes CK, Walhovd KB, Douaud G, Woolrich MW, Westlye LT (2012). Benefits of multi-modal fusion analysis on a large-scale dataset: life-span patterns of inter-subject variability in cortical morphometry and white matter microstructure. Neuroimage.

[41] Marquand AF, Rezek I, Buitelaar J, Beckmann CF (2016). Understanding heterogeneity in clinical cohorts using normative models: beyond case-control studies. Biol Psychiatry.

[42] Heasman B, Gillespie A (2019). Neurodivergent intersubjectivity: distinctive features of how autistic people create shared understanding. Autism.

[43] Joshi G, Wozniak J, Petty C, Martelon MK, Fried R, Bolfek A, Kotte A, Stevens J, Furtak SL, Bourgeois M, Caruso J, Caron A, Biederman J (2013). Psychiatric comorbidity and functioning in a clinically referred population of adults with autism spectrum disorders: a comparative study. J Autism Dev Disord.

[44] Henry CA, Nowinski L, Koesterer K, Ferrone C, Spybrook J, Bauman M (2014). Low rates of depressed mood and depression diagnoses in a clinic review of children and adolescents with autistic disorder. J Child Adolesc Psychopharmacol.

[45] Tebartz van Elst L, Pick M, Biscaldi M, Fangmeier T, Riedel A (2013). High-functioning autism spectrum disorder as a basic disorder in adult psychiatry and psychotherapy: psychopathological presentation, clinical relevance and therapeutic concepts. Eur Arch Psychiatry Clin Neurosci.

[46] Lopez BR, Lincoln AJ, Ozonoff S, Lai Z (2005). Examining the relationship between executive functions and restricted, repetitive symptoms of Autistic Disorder. J Autism Dev Disord.

[47] Barkley RA (2003). Issues in the diagnosis of attention-deficit/hyperactivity disorder in children. Brain Dev.

[48] Lawson RA, Papadakis AA, Higginson CI, Barnett JE, Wills MC, Strang JF, Wallace GL, Kenworthy L (2015). Everyday executive function impairments predict comorbid psychopathology in autism spectrum and attention deficit hyperactivity disorders. Neuropsychology.

[49] Nomi JS, Uddin LQ (2015). Developmental changes in large-scale network connectivity in autism. Neuroimage Clin.

[50] Vaidya CJ, You X, Mostofsky S, Pereira F, Berl MM, Kenworthy L (2020). Data-driven identification of subtypes of executive function across typical development, attention deficit hyperactivity disorder, and autism spectrum disorders. J Child Psychol Psychiatry.

[51] Shapero BG, Gibb BE, Archibald A, Wilens TE, Fava M, Hirshfeld-Becker DR (2021). Risk factors for depression in adolescents with ADHD: the impact of cognitive biases and stress. J Atten Disord.

[52] Gotlib IH, Joormann J (2010). Cognition and depression: current status and future directions. Annu Rev Clin Psychol.

[53] Armstrong T, Olatunji BO (2012). Eye tracking of attention in the affective disorders: a meta-analytic review and synthesis. Clin Psychol Rev.

[54] Vrijsen JN, van Amen CT, Koekkoek B, van Oostrom I, Schene AH, Tendolkar I (2017). Childhood trauma and negative memory bias as shared risk factors for psychopathology and comorbidity in a naturalistic psychiatric patient sample. Brain Behav.

[55] Beck AT, Friedman RJ, Katz MM (1974). The development of depression: a cognitive model. The Psychology of Depression: Contemporary Theory and Research.

[56] Beck AT (2008). The evolution of the cognitive model of depression and its neurobiological correlates. Am J Psychiatry.

[57] De Raedt R, Koster EHW (2010). Understanding vulnerability for depression from a cognitive neuroscience perspective: a reappraisal of attentional factors and a new conceptual framework. Cogn Affect Behav Neurosci.

[58] Mayberg HS (1997). Limbic-cortical dysregulation: a proposed model of depression. J Neuropsychiatry Clin Neurosci.

[59] Phillips ML, Drevets WC, Rauch SL, Lane R (2003). Neurobiology of emotion perception II: implications for major psychiatric disorders. Biol Psychiatry.

[60] Hare TA, Tottenham N, Galvan A, Voss HU, Glover GH, Casey BJ (2008). Biological substrates of emotional reactivity and regulation in adolescence during an emotional go-nogo task. Biol Psychiatry.

[61] Kleinhans NM, Johnson LC, Richards T, Mahurin R, Greenson J, Dawson G, Aylward E (2009). Reduced neural habituation in the amygdala and social impairments in autism spectrum disorders. Am J Psychiatry.

[62] Swartz JR, Wiggins JL, Carrasco M, Lord C, Monk CS (2013). Amygdala habituation and prefrontal functional connectivity in youth with autism spectrum disorders. J Am Acad Child Adolesc Psychiatry.

[63] Wiggins JL, Swartz JR, Martin DM, Lord C, Monk CS (2014). Serotonin transporter genotype impacts amygdala habituation in youth with autism spectrum disorders. Soc Cogn Affect Neurosci.

[64] Fisher PM, Meltzer CC, Price JC, Coleman RL, Ziolko SK, Becker C, Moses-Kolko EL, Berga SL, Hariri AR (2009). Medial prefrontal cortex 5-HT(2A) density is correlated with amygdala reactivity, response habituation, and functional coupling. Cereb Cortex.

[65] Hollocks MJ, Jones CRG, Pickles A, Baird G, Happé F, Charman T, Simonoff E (2014). The association between social cognition and executive functioning and symptoms of anxiety and depression in adolescents with autism spectrum disorders. Autism Res.

[66] De Lissnyder E, Koster EHW, De Raedt R (2011). Emotional interference in working memory is related to rumination. Cogn Ther Res.

[67] Peters AT, Burkhouse KL, Kinney KL, Phan KL (2019). The roles of early-life adversity and rumination in neural response to emotional faces amongst anxious and depressed adults. Psychol Med.

